# Supplementary material for: Hybrid Models and Biological Model Reduction with PyDSTool
Source: PLoS Comput Biol. 2012 Aug 9;8(8):e1002628. doi: 10.1371/journal.pcbi.1002628 (PMC3415397; doi:10.1371/journal.pcbi.1002628)
Supplement: Text S4 — Complete source code for the PyDSTool package (version 0.88.120504). Includes API documentation and help files linking to web pages. This file is identical to the current public release on Sourceforge.net. (ZIP) [file pcbi.1002628.s004.zip › PyDSTool/html/PyDSTool.Points.Pointset-class.html]

xml version="1.0" encoding="ascii"?


PyDSTool.Points.Pointset


| Home | Trees | Indices | Help | PyDSTool |
| --- | --- | --- | --- | --- |

Package PyDSTool ::
Module Points ::
Class Pointset

[hide private]

[frames] | no frames]

# Class Pointset

source code

```
object --+    
         |    
     Point --+
             |
            Pointset
```

---

1D parameterized or non-parameterized set of discrete points. (If
present, the independent variable must be a float64 or an int32)


|  |  |
| --- | --- |
| Instance Methods | [hide private] |

|  |  |
| --- | --- |
| \_\_init\_\_(self, kwd=None, \*\*kw)  x.\_\_init\_\_(...) initializes x; see x.\_\_class\_\_.\_\_doc\_\_ for signature | source code |

|  |  |
| --- | --- |
| \_\_delitem\_\_(self, k)  Remove point by index or by coordinate. | source code |

|  |  |
| --- | --- |
| remove(self, ix)  Remove individual Point by its index. | source code |

|  |  |
| --- | --- |
| reverse(self)  Reverse order of points \*IN PLACE\*. | source code |

|  |  |
| --- | --- |
| rename(self, coord, newcoord)  Rename a coordinate. | source code |

|  |  |
| --- | --- |
| makeIxMaps(self) | source code |

|  |  |
| --- | --- |
| addlabel(self, ix, label, info=None)  Add string label to indexed point. | source code |

|  |  |
| --- | --- |
| removelabel(self, ix)  Remove all labels at indexed point. | source code |

|  |  |
| --- | --- |
| bylabel(self, s)  Return pointset containing points labelled with the supplied labels. | source code |

|  |  |
| --- | --- |
| \_\_setitem\_\_(self, ix, p)  Change individual points, accessed by index (no slicing supported). | source code |

|  |  |
| --- | --- |
| \_\_getitem\_\_(self, ix) | source code |

|  |  |
| --- | --- |
| \_resolve\_indepvar(self, p) | source code |

|  |  |
| --- | --- |
| setTol(self, tol) | source code |

|  |  |
| --- | --- |
| \_\_call\_\_(self, p, coords=None) | source code |

|  |  |
| --- | --- |
| \_\_len\_\_(self) | source code |

|  |  |
| --- | --- |
| \_\_contains\_\_(self, other) | source code |

|  |  |
| --- | --- |
| \_\_lt\_\_(self, other) | source code |

|  |  |
| --- | --- |
| \_\_gt\_\_(self, other) | source code |

|  |  |
| --- | --- |
| \_\_le\_\_(self, other) | source code |

|  |  |
| --- | --- |
| \_\_ge\_\_(self, other) | source code |

|  |  |
| --- | --- |
| \_\_eq\_\_(self, other) | source code |

|  |  |
| --- | --- |
| \_\_ne\_\_(self, other) | source code |

|  |  |
| --- | --- |
| insert(self, parg, ix=None)  Insert individual Point or Pointset before the given index. | source code |

|  |  |
| --- | --- |
| append(self, parg, t=None, skipMatchingIndepvar=False)  Append individual Point or Pointset in place. | source code |

|  |  |
| --- | --- |
| extend(self, parg, t=None, skipMatchingIndepvar=False)  Append individual Point or Pointset in place. | source code |

|  |  |
| --- | --- |
| toarray(self, include\_indepvar=False)  Convert the pointset to a D x L array (one variable per row), where D is the dimension of the pointset and L is its length. | source code |

|  |  |
| --- | --- |
| todict(self, aslist=False)  Convert Pointset to a dictionary of arrays (or of lists with aslist=True). | source code |

|  |  |
| --- | --- |
| \_infostr(self, verbose=0) | source code |

|  |  |
| --- | --- |
| \_\_repr\_\_(self)  str(x) | source code |

|  |  |
| --- | --- |
| \_\_str\_\_(self)  str(x) | source code |

|  |  |
| --- | --- |
| info(self, verboselevel=1) | source code |

|  |  |
| --- | --- |
| \_\_copy\_\_(self) | source code |

|  |  |
| --- | --- |
| copy(self) | source code |

|  |  |
| --- | --- |
| \_\_getstate\_\_(self) | source code |

|  |  |
| --- | --- |
| \_\_setstate\_\_(self, state) | source code |

|  |  |
| --- | --- |
| \_match\_indepvararray(self, other)  Verifies the matching of independent variable arrays in two pointsets. | source code |

|  |  |
| --- | --- |
| \_\_add\_\_(self, other) | source code |

|  |  |
| --- | --- |
| \_\_radd\_\_(self, other) | source code |

|  |  |
| --- | --- |
| \_\_sub\_\_(self, other) | source code |

|  |  |
| --- | --- |
| \_\_rsub\_\_(self, other) | source code |

|  |  |
| --- | --- |
| \_\_mul\_\_(self, other) | source code |

|  |  |
| --- | --- |
| \_\_rmul\_\_(self, other) | source code |

|  |  |
| --- | --- |
| \_\_div\_\_(self, other) | source code |

|  |  |
| --- | --- |
| \_\_rdiv\_\_(self, other) | source code |

|  |  |
| --- | --- |
| find(self, indepval, end=None)  find returns an integer index for where to place a point having independent variable value <indepval> in the Pointset, if <indepval> already exists. | source code |

|  |  |
| --- | --- |
| findIndex(self, indepval, end=None)  find returns an integer index for where to place a point having independent variable value <indepval> in the Pointset, if <indepval> already exists. | source code |

**Inherited from `Point`**:
`__abs__`,
`__neg__`,
`__pos__`,
`__pow__`,
`__rtruediv__`,
`__truediv__`,
`get`,
`has_key`,
`items`,
`iteritems`,
`iterkeys`,
`itervalues`,
`keys`,
`mapNames`,
`update`,
`values`

**Inherited from `Point`** (private):
`_force_coords_to_ixlist`,
`_map_names_to_ixs`

**Inherited from `object`**:
`__delattr__`,
`__getattribute__`,
`__hash__`,
`__new__`,
`__reduce__`,
`__reduce_ex__`,
`__setattr__`


|  |  |
| --- | --- |
| Properties | [hide private] |

**Inherited from `object`**:
`__class__`


|  |  |
| --- | --- |
| Method Details | [hide private] |

|  |  |
| --- | --- |
| \_\_init\_\_(self, kwd=None, \*\*kw)  *(Constructor)* | source code |

x.\_\_init\_\_(...) initializes x; see x.\_\_class\_\_.\_\_doc\_\_ for
signature

Overrides: object.\_\_init\_\_: *(inherited documentation)*

|  |  |
| --- | --- |
| \_\_delitem\_\_(self, k)  *(Index deletion operator)* | source code |

Remove point by index or by coordinate.

Overrides: Point.\_\_delitem\_\_

|  |  |
| --- | --- |
| makeIxMaps(self) | source code |

Overrides: Point.makeIxMaps

|  |  |
| --- | --- |
| addlabel(self, ix, label, info=None) | source code |

Add string label to indexed point. info dictionary is optional

Overrides: Point.addlabel

|  |  |
| --- | --- |
| removelabel(self, ix) | source code |

Remove all labels at indexed point.

Overrides: Point.removelabel

|  |  |
| --- | --- |
| bylabel(self, s) | source code |

Return pointset containing points labelled with the supplied labels.
Argument s can be a string or a list of strings.

|  |  |
| --- | --- |
| \_\_setitem\_\_(self, ix, p)  *(Index assignment operator)* | source code |

Change individual points, accessed by index (no slicing supported).
Individual coordinate values of a point can be changed by adding a
cross-reference coordinate name or index. If ix is a variable name then
the entire row can be changed (again, no slicing supported).

Overrides: Point.\_\_setitem\_\_

|  |  |
| --- | --- |
| \_\_getitem\_\_(self, ix)  *(Indexing operator)* | source code |

Overrides: Point.\_\_call\_\_

|  |  |
| --- | --- |
| \_\_call\_\_(self, p, coords=None)  *(Call operator)* | source code |

Overrides: Point.\_\_call\_\_

|  |  |
| --- | --- |
| \_\_len\_\_(self)  *(Length operator)* | source code |

Overrides: Point.\_\_len\_\_

|  |  |
| --- | --- |
| \_\_contains\_\_(self, other)  *(In operator)* | source code |

Overrides: Point.\_\_contains\_\_

|  |  |
| --- | --- |
| \_\_lt\_\_(self, other)  *(Less-than operator)* | source code |

Overrides: Point.\_\_lt\_\_

|  |  |
| --- | --- |
| \_\_gt\_\_(self, other)  *(Greater-than operator)* | source code |

Overrides: Point.\_\_gt\_\_

|  |  |
| --- | --- |
| \_\_le\_\_(self, other)  *(Less-than-or-equals operator)* | source code |

Overrides: Point.\_\_le\_\_

|  |  |
| --- | --- |
| \_\_ge\_\_(self, other)  *(Greater-than-or-equals operator)* | source code |

Overrides: Point.\_\_ge\_\_

|  |  |
| --- | --- |
| \_\_eq\_\_(self, other)  *(Equality operator)* | source code |

Overrides: Point.\_\_eq\_\_

|  |  |
| --- | --- |
| \_\_ne\_\_(self, other) | source code |

Overrides: Point.\_\_ne\_\_

|  |  |
| --- | --- |
| insert(self, parg, ix=None) | source code |

Insert individual Point or Pointset before the given index.

If ix is not given then the source and target Pointsets must be
parameterized. In this case the Point or Pointset will be inserted
according to the ordering of independent variable values.

|  |  |
| --- | --- |
| append(self, parg, t=None, skipMatchingIndepvar=False) | source code |

Append individual Point or Pointset in place.

skipMatchingIndepvar option causes a matching independent variable
value at the beginning of p to be skipped (only meaningful for appending
parameterized Pointsets). This option is mainly for internal use.

|  |  |
| --- | --- |
| extend(self, parg, t=None, skipMatchingIndepvar=False) | source code |

Append individual Point or Pointset in place.

skipMatchingIndepvar option causes a matching independent variable
value at the beginning of p to be skipped (only meaningful for appending
parameterized Pointsets). This option is mainly for internal use.

|  |  |
| --- | --- |
| toarray(self, include\_indepvar=False) | source code |

Convert the pointset to a D x L array (one variable per row), where D
is the dimension of the pointset and L is its length. (This is a copy of
the internal attribute 'coordarray'.)

If the optional include\_indepvar switch is set True (default False),
the first row is the independent variable, and the whole array will be
(D+1) x L in shape.

Overrides: Point.toarray

|  |  |
| --- | --- |
| todict(self, aslist=False) | source code |

Convert Pointset to a dictionary of arrays (or of lists with
aslist=True).

Overrides: Point.todict

|  |  |
| --- | --- |
| \_infostr(self, verbose=0) | source code |

Overrides: Point.\_infostr

|  |  |
| --- | --- |
| \_\_repr\_\_(self)  *(Representation operator)* | source code |

str(x)

Overrides: object.\_\_repr\_\_: *(inherited documentation)*

|  |  |
| --- | --- |
| \_\_str\_\_(self)  *(Informal representation operator)* | source code |

str(x)

Overrides: object.\_\_str\_\_: *(inherited documentation)*

|  |  |
| --- | --- |
| info(self, verboselevel=1) | source code |

Overrides: Point.info

|  |  |
| --- | --- |
| \_\_copy\_\_(self) | source code |

Overrides: Point.\_\_copy\_\_

|  |  |
| --- | --- |
| copy(self) | source code |

Overrides: Point.\_\_copy\_\_

|  |  |
| --- | --- |
| \_\_getstate\_\_(self) | source code |

Overrides: Point.\_\_getstate\_\_

|  |  |
| --- | --- |
| \_\_setstate\_\_(self, state) | source code |

Overrides: Point.\_\_setstate\_\_

|  |  |
| --- | --- |
| \_match\_indepvararray(self, other) | source code |

Verifies the matching of independent variable arrays in two pointsets.
Does nothing if either object is not a parameterized pointset.

|  |  |
| --- | --- |
| \_\_add\_\_(self, other)  *(Addition operator)* | source code |

Overrides: Point.\_\_add\_\_

|  |  |
| --- | --- |
| \_\_radd\_\_(self, other)  *(Right-side addition operator)* | source code |

Overrides: Point.\_\_add\_\_

|  |  |
| --- | --- |
| \_\_sub\_\_(self, other)  *(Subtraction operator)* | source code |

Overrides: Point.\_\_sub\_\_

|  |  |
| --- | --- |
| \_\_rsub\_\_(self, other) | source code |

Overrides: Point.\_\_rsub\_\_

|  |  |
| --- | --- |
| \_\_mul\_\_(self, other) | source code |

Overrides: Point.\_\_mul\_\_

|  |  |
| --- | --- |
| \_\_rmul\_\_(self, other) | source code |

Overrides: Point.\_\_mul\_\_

|  |  |
| --- | --- |
| \_\_div\_\_(self, other) | source code |

Overrides: Point.\_\_div\_\_

|  |  |
| --- | --- |
| \_\_rdiv\_\_(self, other) | source code |

Overrides: Point.\_\_rdiv\_\_

|  |  |
| --- | --- |
| find(self, indepval, end=None) | source code |

find returns an integer index for where to place a point having
independent variable value <indepval> in the Pointset, if
<indepval> already exists. Otherwise, a pair indicating the nearest
independent variable values present in the Pointset is returned.

To ensure an integer is always returned, choose a left or right side
to choose from the pair, using end=0 or 1 respectively.

|  |  |
| --- | --- |
| findIndex(self, indepval, end=None) | source code |

find returns an integer index for where to place a point having
independent variable value <indepval> in the Pointset, if
<indepval> already exists. Otherwise, a pair indicating the nearest
independent variable values present in the Pointset is returned.

To ensure an integer is always returned, choose a left or right side
to choose from the pair, using end=0 or 1 respectively.

  


| Home | Trees | Indices | Help | PyDSTool |
| --- | --- | --- | --- | --- |

|  |  |
| --- | --- |
| Generated by Epydoc 3.0.1 on Fri May 4 15:24:07 2012 | http://epydoc.sourceforge.net |
